# The sero-prevalence of brucellosis in cattle and their herders in Bahr el Ghazal region, South Sudan

**DOI:** 10.1371/journal.pntd.0006456

**Published:** 2018-06-20

**Authors:** Nuol Aywel Madut, Adrian Muwonge, George William Nasinyama, John Bwalya Muma, Jacques Godfroid, Ambrose Samuel Jubara, James Muleme, Clovice Kankya

**Affiliations:** 1 Department of Clinical Studies, Faculty of Veterinary Science, University of Bahr el Ghazal, Wau, South Sudan; 2 Department of Biosecurity, Ecosystems &Veterinary Public Health (BEP), College of Vet. Animal Resources & Biosecurity (COVAB), Makerere University, Kampala, Uganda; 3 Div. Genetics and Genomics, The Roslin Institute, Royal (Dick) School of Veterinary Studies, University of Edinburgh, Easter Bush, Midlothian, United kingdom; 4 Department of Disease Control, School of Veterinary Medicine, University of Zambia, Lusaka Zambia; 5 Department of Arctic and Marine Biology, University of Tromsø, Tromsø, Norway; Yale University Yale School of Public Health, UNITED STATES

## Abstract

**Background:**

Brucellosis is a worldwide recognized bacterial zoonotic disease. There is currently no information on bovine brucellosis sero-prevalence in South Sudan regardless of the economic, social and public health impact on populations. Therefore, for the first time in 33 years, we report the sero-prevalence of brucellosis in cattle and their herders. Furthermore, we characterize the drivers associated with the disease at the human-animal interface in Bahr el Ghazal region, South Sudan.

**Methods:**

A total of 893 and 87 animal and human sera respectively were examined between December 2015 and May 2016. Rose Bengal Plate Test (RBPT) and Competitive Enzyme Linked Immuno Sorbent Assay (c-ELISA) were used in parallel to detect anti-*Brucella* antibodies. Questionnaires were administered to collect relevant metadata used for the association analysis in R version 3.2.3. Odds Ratio (OR) and Confidence Intervals (CI) were determined.

**Results:**

Overall bovine brucellosis prevalence was 31% (95%CI = 28.0–34.2), with the highest 63% (95%CI = 53–70) and lowest 10% (95%CI = 4.5–20.1) prevalence estimates in Wau and Gogrial states respectively. The bovine sero-prevalence was approximately equally distributed among the male 30.4% (26.9–34.2) and the females 32.5% (26.8–38.7). Poor body condition (OR = 0.22; 95%CI = 0.07–0.54) and larger herd sizes (OR = 0.05; 95%CI = 0.008–0.173) were protective factors for brucellosis, while the opposite was true for the second (OR = 1.70; 95%CI = 1.08–2.67) and third (OR = 2.5; 95%CI = 1.46–4.47) lactation stage. The overall brucellosis sero-prevalence in herders was estimated at 33.3% (23.9–44.3).

**Conclusion:**

We report a high prevalence of anti-*Brucella* antibodies in cattle and their herders in Bahr el Ghazal, indicating an enzootic status in the cattle population being an important source of infection for humans. This represents a genuine public health challenge. Therefore, there is need to raise awareness and build capacity and infrastructure in this fragile state to underwrite future public health strategies for brucellosis.

## Author summary

Enzootic brucellosis is a bacterial infectious disease, which represents millions of dollars in production losses in livestock, as well as Disability- Adjusted Life Years (DALYS) associated with health and treatment in the human populations for Low and Middle Income Countries (LMICs). Despite these unequivocally known challenges, there has not been any reporting on this disease in livestock and humans in the recent past. The limitations the disease puts on livestock health and production, coupled with the zoonotic form, which is primarily driven by risky food consumption habits, and interaction with livestock in an enzootic setting with uncontrolled animal movement, means that this disease is not only of veterinary public health, but of trans-boundary importance as well.

## Introduction

Brucellosis is described as a highly contagious zoonotic disease, and a cause of significant reproductive losses in livestock [1]. Brucellosis is common in Low and Middle Income Countries (LMICs), characterized by poor hygiene, consumption of raw animal products (like milk and meat), and lack of public health education programs[2, 3]. Animal brucellosis causes direct socio-economic effects in communities dependent on animal production as their livelihood. Losses in animals are attributed to loss of offspring due to abortion, stillbirth and infertility. Indirect losses are due to reduction in milk yields and humans suffering from the disease. Bovine brucellosis is caused mainly by *Brucella abortus*, although *Brucella melitensis* may spillover from the small ruminant reservoir and infect the cattle too. On the other hand, *Brucella suis* infects pigs, as well as humans [4].

In LMICs, the prevalence of animal and human brucellosis is generally unknown due to a myriad of challenges with diagnostics, reporting and weak to non-existent surveillance systems, especially in malaria endemic areas [5, 6]. In Africa, brucellosis is an enzootic disease in livestock [7, 8]. In South Sudan, brucellosis prevalence is unknown due to the lack of awareness among communities about the disease, but more importantly due to weakened animal and public health systems as a result of political and civil instability. As a consequence, the disease remains largely neglected with little attention given to prevention and control in livestock and humans. Livestock, especially cattle, is the main source of livelihood for communities in Bahr el Ghazal region [9, 10], which inherently increases their risk to zoonotic diseases [11]. Animal ownership has for long been documented as the main risk for exposure to *Brucella spp* infection through direct contact with infected animal material and consumption of raw milk and infected meat[2].Therefore, documenting the risk profile at the human-cattle interface in such settings is central to developing control strategies in such a setting. Here, we estimate the sero-prevalence of brucellosis in cattle and their herders, as well as characterize the drivers associated with the disease at the human-animal interface in cattle camps of Bahr el Ghazal region, South Sudan.

## Materials and methods

### Study design

This was a cross-sectional study of herders and their cattle conducted between December 2015 and May 2016 in the Greater Bahr el Ghazal region. We collected quantitative data and metadata (socio-demographic data and animal attributes) using a structured questionnaire.

### Study setting

**Fig 1:**
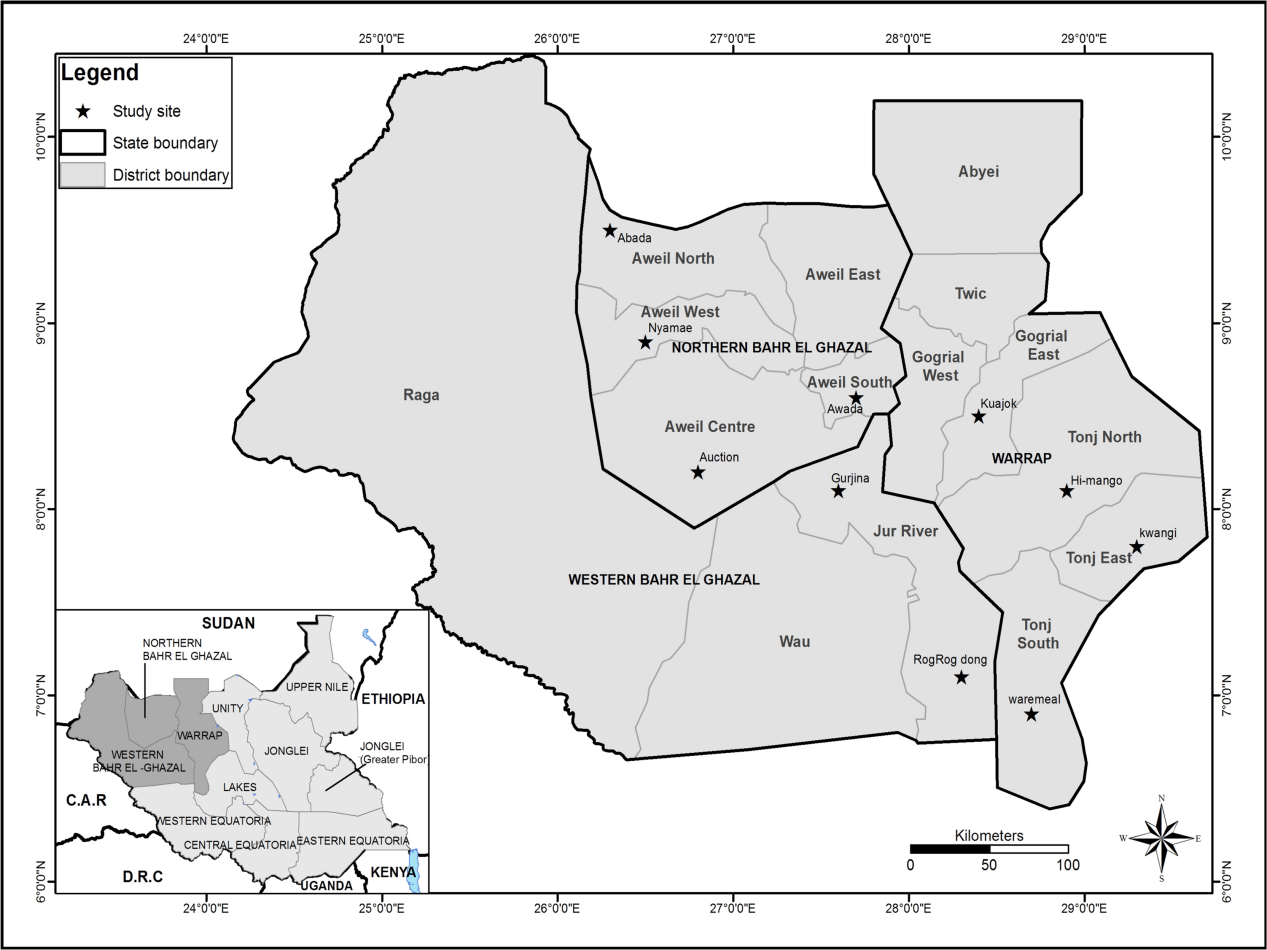
The map of Greater Bahr el Ghazal showing all the ten states. The map was generated using cartographic data from World Geodetic System 1984 (WGS 84) (https://confluence.qps.nl/pages/viewpage.action?pageId=29855173) Black diamond- represents the location of the cattle camps where our sampling was conducted.

Greater Bahr el Ghazal region is situated in the Northwestern part of South Sudan. The region consists of ten states, Aweil, Aweil East, Gogrial, Lol, Tonj, Twic, Wau, Gok, Eastern Lakes, and Western Lakes. The region consists of vast land with iron plateau and swamps feeding 12 million heads of cattle which represents 50% of the National herd [12, 13]. It is predominantly inhabited by the Dinka ethnic group, who are cattle herders; other ethnic groups like Balanda and Kerash mostly practice agro-pastoralism [2]. This mixture of land use allows for complex human-animal interactions usually compounded by the high population density [13]. It is these complex dynamics that our study was aiming to unravel with respect to brucellosis. A map of our study area is shown in Fig 1.

### Study population

The study population consisted of cattle and herders from cattle camps in Bahr el Ghazal region. The cattle belonged to herders in the areas of Aweil, Gogrial, Tonj, and Wau states. Majority of cattle is owned by pastoralists who migrate throughout the dry season looking for pastures in small groups of families or in large groups of villages [12]. In effect, the herds in each grouping can be owned by more than one family, but usually it is a herd per family. The herdsmen who tend to the animals are usually relatives of the owners, but in rare circumstances, families can employ herdsmen to manage their cattle herds.

### Sample size

The sample size for cattle was estimated using a bovine brucellosis prevalence (6.5%) previously reported in Greater Bahr el Ghazal [13]. We assumed a brucellosis test sensitivity and specificity of 85%, and 90% respectively [14], and a precision 0.05 with 95% confidence intervals. We would then be expected to sample 346 cattle, however, 893 samples were collected using a systematic random sampling procedure, which allowed us to improve the precision of the sero-prevalence estimates [15].

Since herds are owned and maintained in cattle camps, large numbers of cattle tend to be clustered in fewer cattle camps. In this regard, we conveniently screened 87 herders from 37 cattle camps in two of the four states (Tonj and Aweil). Note that the screening was done with consent from the herders, as described in the ethical consideration section.

### Sampling procedure

The four states where the study was conducted were purposively selected basing on the safety of the area at the time of the study. Between one and three cattle camps were selected per state, for each cattle camp; lists of herders with their respective herds were obtained from the veterinary office. Usually, a camp contained up to 100 herds each with on average 150 animals. We randomly selected thirty-percent of the herds in each camp. This was done by dividing the number of herds on the camp-herd list by thirty, and the quotient was used as the interval for selecting the herds from the camp-herd list. For the selected herds, we then sought permission from the herd owner and only if granted, would we ask for the number of animals in their herds. This number would then be divided by 10, and the quotient was used as an interval for selecting individual animals lined up in a kraal. If the herd owner rejected our request, the herd was dropped and we continued with same frequency as before, in order to get a replacement herd. A total of 893 animals were selected from 37 cattle camps from four the states. It is noteworthy that we selected a minimum of 20 animals per herd using this strategy.

We also collected blood samples from the herders who were in direct contact with animals and were willing to participate in this study according to the informed consent. However, it is important to note that some of the herders were below eighteen years; in this case we sought permission from their parents and guardian.

### Data collection

Information about animal body condition status, history of abortion and presence of hygroma as indicators of brucellosis were captured on data sheet during blood collection by observation and interviewing animal’s owners. In addition, information on herd size, lactating stage and age of the animal was collected. Age was determined using the dentition method of ageing cattle. From all the participants, a questionnaire was administered, and we collected information about the individual’s occupation, age, sex, marital status and education level.

### Sample collection

Ten (10) ml of blood were collected from the jugular vein of the selected animal by a veterinary research assistant; on the other hand, 5 mL of blood was collected from the cephalic vein of each herder by a registered nurse. All blood samples were then kept at room temperature (25°C-30°C), and tilted at an angle of 45° for 6–8 hours to allow for clotting. The sera were aliquoted into new set of labeled Eppendorf tubes, stored on ice packs and transported to Wau Teaching Hospital Laboratory, where they were kept in a deep freezer at -80°C. The samples were then transported to the Central Diagnostic Laboratory at Makerere University, College of Veterinary Medicine and Biosecurity, Kampala-Uganda by air after completion of data collection. Here, serological tests that included, Rose Bengal Plate Test (RBPT), and Competitive Enzyme Linked Immuno sorbent Assay (c-ELISA) were done within five days of delivery as described below.

### Serological testing

#### Rose Bengal plate test (RBPT)

The antigen used in the RBPT was obtained from the Veterinary Research Institute, Sudan. It was prepared and standardized following the procedure as described by Al ton [16]. The antigen was quality controlled according to the OIE guidelines (2016) [17]. The same secondary standard sera were used as positive and negative controls during the testing. Prior to testing, serum samples and the antigen were removed from the refrigerator and left to thaw at room temperature. Following equilibration of serum at room temperature, 30μL of serum was dispensed into an enamel plate, an equal volume of RBPT antigen was also added to the plate, and then mixed. After rocking the plate for 5 minutes, the test result was read. Agglutination was recorded as negative (0), weak positive (+1), positive (+2), strong positive (+3) or very strong positive (+4) as earlier described[16]. The plates with negative and weakly positive agglutination were considered negative. Positive and negative control sera were run in parallel for each sera batch tested. Duplicates of each tested serum were used to assure that the antigens used in the test were sensitive, as well as specific [18].

#### Competitive Enzyme linked immune sorbent assay (c-ELISA)

The test kit, Serum P04130-13 brucellosis Antibody Test Kit–IDEXX was used as a confirmatory test. The test was performed following the manufacturer’s guidelines. Briefly, PBS-Tween Solution 20x concentrate 1/20 was diluted in distilled water. Then, 500 mL were prepared by adding 25ml PBS-Tween Solution to 475 mL distilled water and mixed thoroughly. After this, freeze dried mAb was reconstituted immediately before use with 6mL sample dilution buffer by adding the buffer carefully into the bottle. All reagents were equilibrated to room temperature (21°C-28°C) for 30–45 minutes before use. The samples and controls were diluted by adding them to the wells of the plates prefilled with the buffer. Fifty-microliter of each serum controls (positive, weak positive and negative) were added into each of the appropriate wells, respectively. Each control was run in duplicate. Five-microliter of each sample dilution buffer was added into two separate wells (designated as conjugate control). Five-microliter of each test sample was added to each appropriate well, and then 50μL of mAb-solution were added to all wells of the samples and controls within 10 minutes. The test plate was sealed and the reagents were mixed thoroughly by tipping the sides of the plate. The plate was incubated at room temperature for 30 minutes. Thereafter, the plate was rinsed 4 times with PBS-Tween using Sanofi Pasteur washer. One hundred microliters (100-μl) substrate solutions were added to each well and incubated for 10 minutes at room temperature; timing began after the first well was filled. The reaction was stopped by adding 50μL of 4% sodium dodecyl sulphate (SDS), in the same order as the substrate solution was added to each well and mixed thoroughly. The optical densities (OD) of the controls and the test samples were measured using a micro plate photometer (Bio Tek ELX800 absorbance reader) at 450 nm after 15 minutes from the addition of the stop solution. The mean and standard deviations of the optical densities of the test and control samples were calculated. The percentage inhibition (PI) for the controls and test samples were calculated using a formula (PI = 100 –(Mean OD of test sample/ Mean OD of Controls X 100)). To ensure quality, the OD values of the control and test samples were required to fall within 0.75–2.0. PI values <30% were considered negative and those > 30% are classified positive. The serological tests were performed following the OIE guidelines as specified in Diagnostic Tests and Vaccines for Terrestrial Animals, chapter 2.1.4 (OIE,2016)

### Data analysis

Statistical analysis was done in SPSS and R, version 24 and 3.2.3 respectively. For descriptive statistics, that is to say, proportions and percentage of the positive against the number tested were estimated; while Chi-square was employed in assessing the relationship between various factors and test outcome (test positivity). A positive *Brucella* sample was defined as sample that was positive on RBPT and confirmed by c-ELISA, while a negative sample was defined as a sample that was negative on RBPT, as well as c-ELISA. A logistic regression model was developed to identify factors associated with bovine brucellosis in cattle. The model developed by adding variables in a forward selection process adjusting for confounding, starting with variables that had the lowest p-value from the univariable analysis. Only variables that had a p value <0.25 were included in the model, these were added and removed to see if they still retained their level of statistical significance (p < 0.05), and checked for potential confounding effects as well. The least complex model was chosen based on the lowest Akaike information criterion (AIC). Standard post estimation statistics were also done.

### Ethics considerations

This study involved an administration of questionnaires to the herders, as well as blood sampling from cattle. Therefore, the study protocol (SBLS/REC/15/133) was assessed and approved by the Ethical Review Committee of the College of Veterinary Medicine, Animal Resources and Biosecurity (COVAB), Makerere University, Uganda; reference number SBLS.NA.2015 (S1). We also obtained permission to collect human and animal samples from Ministry of Health (MOH) (S2), and Ministry of Agriculture, Animal Industry and Fisheries (MAAIF)—RSS/MLFI/DVS/J/15/7 (S3), South Sudan. Furthermore, we sought consent from the participants in this study. Their decision to participate was arrived at, after we explained the objectives and the potential benefits of this work to them as individuals and their communities at large. For individuals who were below 18 years of age, we sought permission from their parents or guardians. All this was done in local ethnic language of the group, to which the individual belonged. All this was in addition to the assurance of anonymity as required by the ethical approval obtained. We also obtained import and export permits for biological sample transportation from Ministries of Agriculture of Uganda and South Sudan (S4& S5).

## Results

### Descriptive statistics for bovine brucellosis

A total of 893 serum samples were examined in the study; 138, 70, 198, and 487 from Wau, Gogrial, Tonj and Aweil, respectively. We sampled 644 female and 249 male cattle with an estimate median age of cattle being seven years (Table 1). Our sero-prevalence estimates are based on c-ELISA, but we estimate a 98% (95% CI = 97–100) kappa agreement between the two tests (Supplementary R code). The overall estimate of bovine brucellosis sero-prevalence was 31.0% (95%CI = 28.0–34.2), which varied by states, the highest and lowest recorded in Wau 63% (95%CI = 53–70) and Gogrial 10% (95%CI = 4.5–20.1) respectively. We observe that the sero-prevalence increased with age; 26% (95%CI = 21.8–32.4) among the young, and 44.2% (95%CI = 37.3–51.4) among the old. The same trend was generally true for herd size (Table 1). There was indication of an association between bovine brucellosis sero-prevalence and age, herd size, body condition status, lactation stage and presence of hygroma and abortion history based on the univariable analysis (Table 1).

**Table 1:** Shows the summary statistics for bovine brucellosis, sero-prevalence estimates and a univariable analysis of social demographic factors in Bahr el Ghazal.

Variable	Level	Total	RBPT(+n)	cELISA (+n)	X^2^	P-value	Prevalence* 95%CI
State	Wau	138	91	87	91.39	2.2e-16*	63(53–70)
	Gogrial	70	7	7	-	-	10(4.5–20.1)
	Tonj	198	65	65	-	-	32.8(26.4–39.9)
	Aweil	487	118	118	-	-	24.2(20.5–28.3)
Age	0–5	283	76	76	22.03	1.64e-05*	26.8(21.8–32.4)
	6–10	411	114	113	-	-	27.5(23.2–32.1)
	11–17	199	91	88	-	-	44.2(37.3–51.4)
Sex	Female	644	198	196	0.394	0.53	30.4(26.9–34.2)
	Male	249	83	81	-	-	32.5(26.8–38.7)
Herd size	< 30	10	7	6	98.93	2.2e-16*	60.0(27.3–86.3)
	30–49	710	165	165	-	-	23.2(20.2–26.5)
	50–100	128	84	81	-	-	63.2(54.2–71.5)
	> 100	45	25	25	-	-	55.5(40.1–70.0)
Lactating Stage	Dry	353	91	90	16.42	0.00093*	25.5(21.1–30.4)
	L1	42	10	10	_	_	23.80(12.6–39.8)
	L2	165	56	56	_	_	33.9(26.8–41.7)
	L3	86	41	40	_	_	46.5(35.7–57.5)
Body condition	Good	816	254	261	18.91	7.8e-05*	31.9(28.8–35.3)
	Poor	57	5	5	_	_	8.7(3.2–20.0)
	Cachectic	20	12	11	_	_	55.0(32.0–76.2)
**Hygroma	No	882	273	269	7.2341	0.007153*	30.5(27.5–33.7)
	Yes	9	7	7	-	-	77.7(40.2–96.1)
History of abortion	No	745	192	192	8.32	0.003927*	25.7(22.7–29.1)
	Yes	29	15	15	-	-	51.7(32.8–70.1)

**Hygroma (n = 891/893) because we did not find information on hygroma for two (2) animals while we were collecting data, whereas for History of abortion (774) because heifers and calves were not included while measuring this variable

* represents factors that are significantly associated with the sero-positive status. Lactation stage: Dry = a period 2 months before calving when the cow is not milked, L1 = period 0-3months after birth, L2 = period from 3 months to 6 months and L3 = period from 6 months to 1 year during which the cow is milked.

### Bovine brucellosis and associated risk factors

Indeed, after taking into account the variation due to all chosen factors, we still observe that herd size, lactation stage and body condition status are still significantly in association with brucellosis sero-prevalence in this area. We observe that other than Lactation stage, the rest of the herd factor seems to be protective for brucellosis sero-prevalence (Table 2).

**Table 2:** Multivariable logistic regression model for brucellosis sero-prevalence and associated herd management factors in Bahr el Ghazal region, South Sudan.

Factors	Description	Odds Ratio at (97.5%)CI	P- value
Lactating stage	Non-lactating	1	Ref
	L1	1.71(0.67–4.08)	0.232
	L2	1.70(1.08–2.67)	0.019
	L3	2.5(1.46–4.47)	0.0008 ***
Herd size	<50	1	Ref
	50–100	0.18(0.03–0.76)	0.03*
	>100	0.05(0.008–0.17)	3.63e-05 ***
History of abortion	No	1	Ref
	Yes	0.46(0.11–1.63)	0.25
BCS	Good	1	Ref
	Poor	0.22(0.07–0.54)	0.002***
	Cachectic	0.21(0.03–1.19)	0.08

BCS = body condition score, Level of statistical significance

*** = 0.001

* = 0.05, AIC = 673.42 and AUC = 0.68

### Zoonotic brucellosis descriptive statistics

A total of 87 cattle herders were recruited in the study from Aweil (n = 40) and Tonj (n = 47) states. Our sample size contained more males, majority of who were between 16–60 years of age and were illiterate. Overall, we estimate the sero-prevalence of zoonotic brucellosis was estimated to be 33.3% (23.9–44.3). The sero-prevalence was comparable between states and occupational activities, but appears to increase by age (Table 3).

**Table 3:** Shows the summary descriptive statistics for zoonotic brucellosis among herders in Bahr el Ghazal.

Variable	Level	Total	RBPT (+n)	cELISA (+n)	X^2^	P-value	Prevalence* 95%CI
State	Tonj	40	18	12	2.08	0.14	30.0(17.1–46.7)
	Aweil	47	17	17	-	-	36.2(23.1–51.5)
Age	6–15*	15	3	3	2.27	0.32	20.0(5.3–48.6)
	16–35	49	16	21	-	-	42.9(29.1–57.7)
	36–60	23	10	11	-	-	47.8(27.4–68.9)
Gender*	Female	62	22	18	1.18	0.27	29.0(18.6–42.1)
	Male	25	13	11	-	-	44.0(25.0–64.7)
Literacy	Illiterate	83	32	26	0.81	0.36	31.3(21.8–42.5)
	Literate	4	3	3	-	-	75.0(21.9–98.6)
Marital Status*	Single	18	3	3	1.97	0.16	16.6(4.4–42.2)
	Married	69	32	26	_	_	37.6(26.5–50.2)
Occupational activity*	Herder	33	12	12	0.01	0.91	36.3(20.9–54.8)
	Milkers	46	20	15	_	_	32.6(19.9–48.3)

6–15* It is noteworthy that information for six of the individuals in this age category will be redundant for the marital status and literacy categories. Eight individuals did not specify their *occupational activity

## Discussion

Human and livestock are inextricably linked in LMICs, which is why the United Nations recognizes the critical role livestock play in the livelihood of people in LMICs [19]. The close proximity, cultural based food consumption habits, poor public health infrastructure and high prevalence of zoonotic disease make this relationship risky for human population in these settings [20]. This is why the World Health Organization also emphasizes the public health challenges of brucellosis in communities where it is enzootic in animal populations [21]. Any sustainable steps towards developing a cost effective public health control strategy can only be underwritten by knowledge of the disease prevalence, and the risk profile that defines its epidemiology. For this matter, we aimed at estimating the sero-prevalence of brucellosis in cattle and their herders in Bahr el Ghazal region, South Sudan, as well as characterizing the risk profile of bovine brucellosis. This output would then form the foundation for efforts controlling the disease in this new independent country.

We estimate a 31% sero-prevalence of bovine brucellosis in the cattle camps of Greater Bahr el Ghazal, South Sudan. Importantly, there is a very good kappa agreement 98% (95% CI = 97–100) between the results; the RBPT and the c-ELISA [22]. We observed the same for the results from the herders in this area, which suggests that in a setting where brucellosis is enzootic at a high sero-prevalence, there is no need to confirm RBPT results by c-ELISA, or vice-versa. Our sero-prevalence estimate is higher than what has been reported in Cameroon (15.9%) [23], and Ethiopia (4.9%) [24], but lower than estimates in Zambia (42%) [25], Nigeria (84.9%) [27] and Uganda (100%) [28]. A sero-prevalence study in Greater Bahr el Ghazal was last conducted 33 years ago [13], and the prevalence observed in the current study is five times higher than the estimates reported then [13]. Although there is a slight difference in diagnostic tests used, the robust sample size used in both studies still renders this comparison valid [29]. The difference in prevalence could be due to a growth in cattle population over time [30], combined with a break down in animal management infra-structure due to political and social instability[12]. In the current study, the highest sero-prevalence (63.0%) was observed in Wau, which likely reflects how herd size contributes to brucellosis transmission and occurrence [31]. These large cattle herds are inherently associated with seasonal cattle movements, which is reported to be a risk factor for exposure [32]. Furthermore, large herd management in such settings requires that bulls are shared to enhance genetic diversity, another documented herd level risk factor [33].This is however contrary to the results of our logistic regression model which suggests that larger herds were a protective variable for bovine brucellosis in Greater Bahr el Ghazal. It is impossible to fully assess what this finding represents, but it is likely associated to the dilution factor that comes with large herds, that is to say; the probability of selecting a positive case is lower in a larger herd [34]. Although the prevalence reported in large herds is lower than what has been reported elsewhere (15.8%) in South Sudan [35], the general distribution of the disease among large herds is comparable in South Sudan [2, 36, 37–39].

A number of studies in Africa have documented sex, breed and age as risk factors of brucellosis at individual and herd level [33]. In this study, we observe an association between lactation stage and bovine brucellosis. A cow in the second or third lactation stage was 1.7 and 2.5 times more likely to be sero-positive for bovine brucellosis than one in the first or dry lactations stage. These findings are in agreement with reports in India and Zimbabwe, where the risk of brucellosis increased in pluriparous cows [27, 40]. Similarly, the phenomenon with age has been extensively documented elsewhere [13], which has been attributed to resistance that exists due to sexual immaturity and or passive immunity of calves acquire to transfer of maternal antibodies through colostrum [41].

From a management point of view, it is the older animals that are usually moved from one place to another during the dry season, which increases their likelihood of contact with other animals, and thus exposure[8,7, 42, 43]. The study also found that poor body condition was a protective factor for brucellosis in these settings, such a finding seems counterintuitive and probably an indirect effect of a variable that was not recorded. The reason for this association could be that animals that were not trekked in search of water and pasture were likely not to come in contact with other infected herds and therefore remain unexposed, but this then came with a penalty inform of malnutrition. Therefore, not going to search for food implied loss of body condition, but reduced risk of exposure likely to result from comingling with other animals. A similar observation has been reported in the Kafue basin of Zambia [44], in that study, animals that resided in the communal grazing areas of the Kafue flats had generally better body condition, but also recorded higher *Brucella* prevalence compared to those that grazed poor pastures around the homesteads.

The existence of brucellosis in the region coupled with the lack of control measures, especially in the traditional sector that maintains the vast majority of animal wealth in Sudan and South Sudan poses economic and public health risk to the communities. Zoonotic brucellosis was estimated at 33.3%(23.9–44.3), which is higher than that reported in a recent studies by Zein in Northern state of Sudan (24.5%)[45], and in Khartoum state (3.9%) among herders [46].

The estimates were also higher than reports in Togo (0.44%) [47]; Tanzania (5.5%)[26]; Chad (3.9%)[48]; and Ethiopia (10.4%)[49]. The prevalence in this study was however lower than the estimate reported on milkers in Khartoum state (40.0%) ten years ago [50]. The prevalence was relatively higher in males 44.0% (25.0–64.7) compared to their female counterparts 29.0% (18.6–42.1). The high overall sero-prevalence observed in the current study represents a huge public health challenge for the meager public health infrastructure in this setting. This is however unlikely to change, unless the current political stand is resolved.

The difference in sero-prevalence observed at gender level could be attributed to the differences in social roles, for example; men spend more time with the animals during herding. A recent study done under the auspices of the WHO highlights the equal contribution of direct contact to animals and the food borne transmission of *Brucella* infections in LMICs [51]. Factors like herding, cleaning and milking in the cattle camps, without using Personal Protective Equipment, and lack of knowledge about zoonotic diseases in general, and brucellosis in animals could be significant to human infection [8]. Helping animals in delivery, removing retained placenta, consumption of raw milk and meat, and using urine for washing hands and utensils, were well known in the region. All these routes represent genuine possibility of transmission in cattle camps of Greater Bahr el Ghazal [2].

### Limitations encountered in this study

Some of the limitations were; security instability in the region due to the civil war and cattle raiding, and the refusal by most of the herders to participate in the study alleging that sampling from cattle would affect their productivity. Moreover, cattle camps were distributed in vast area and difficult to reach.

### Conclusion

We report a high sero-prevalence of brucellosis in cattle in Bahr el Ghazal indicating an enzootic status. Individual animal and herd management factors are linked to the prevalence in animals. This represents a genuine public health challenge underpinned here by the high sero-prevalence estimates in herders. Control of brucellosis in livestock through approved strategies such as vaccinations either with S19 or RB51 reduces the likelihood of a transmission event from animal-animal. This over time can have the result of lower incidence of brucellosis, thus decreasing the spill over into human populations. Furthermore, there is therefore need to build capacity and infrastructure in veterinary delivery services in this fragile state to underwrite future veterinary public health strategies for controlling brucellosis in livestock and mitigating transmission to humans.

## Supporting information

S1 Ethical Approval Approval from Makerere University Institutional Review Board. (PDF)

S2 Ethical Approval Approval from ministry of health (MOH), South Sudan to collect human and animal samples. (PDF)

S3 Ethical Approval Approval from Ministry of Livestock and Fisheries South Sudan. (PDF)

S4 Ethical Approval Import Permit from Ministry of Agriculture, Animal Industry and Fisheries (MAAIF) Uganda. (PDF)

S5 Ethical Approval Export permit from Ministry of Livestock and Fisheries Industry, South Sudan. (PDF)

S1 Dataset Human dataset used for analysis. (SAV)

S2 Dataset Animal dataset used for analysis. (SAV)

S3 Dataset Model summaries. (HTML)
