# Supplementary material for: The sero-prevalence of brucellosis in cattle and their herders in Bahr el Ghazal region, South Sudan
Source: PLoS Negl Trop Dis. 2018 Jun 20;12(6):e0006456. doi: 10.1371/journal.pntd.0006456 (PMC6010255; doi:10.1371/journal.pntd.0006456)
Supplement: S1 Ethical Approval — (PDF) [file pntd.0006456.s001.pdf]

MAKERERE UNIVERSITY
COLLEGE OF VETERINARY MEDICINE,
ANIMAL RESOURCES & BIOSECURITY

OFFICE OF THE DEAN

SAFER PLANET, PROSPEROUS COMMUNITIES

SCHOOL OF BIOSECURITY, BIOTECHNICAL AND LABORATORY SCIENCES (SBLS)

P.O. Box 7062 Kampala, Uganda
Tel : +256-414-554685
Fax : +256-414-554685

Cables : "MAKUNIKA"
Email : sbls@vetmed.mak.ac.ug
Website : www.covab.mak.ac.ug

Your Ref: *Our Ref:* **SBLS.NA.2015** *Date:* **14/12/2015**

RESEARCH AND ETHICS COMMITTEE REVIEW AND ENDORSEMENT

Statement from the Institutional Ethical Review Board:

The REC only accepts for review and approval, research proposals that have been found both scientifically and ethically acceptable in accordance with guidelines on Institutional Ethical Review Boards.

We the **Institutional Ethical Review Committee** established by

COLLEGE OF VETERINARY MEDICINE, ANIMAL RESOURCES AND BIOSECURITY

do certify that we have reviewed the research proposal **(SBLS/REC/15/133)** entitled;

Brucellosis at Human-Domestic Animal Interface in Greater Bahr Elghazal States, South Sudan submitted by

Dr. Nuol Aywel Madut Yajj, Makerere University

We attest to scientific and ethical merit of this study and competency of the investigator(s) to conduct the research and hereby recommend the proposal to the Uganda National Council for Science and Technology (UNCST) for approval

SIGNATURES

	Name	Signature	Date
**Ethics Committee Representative**	Assoc. Prof. Clovice Kankya	[signature]	14/12/2015
**Head of Ethics Committee**	Assoc. Prof. Frank N. Mwiine	[signature]	14/12/2015

CONTACTS

Tel: +256 787 495 220

E-mail: mwiine@covab.mak.ac.ug
fmwiine@gmail.com

OFFICIAL STAMP OF INSTITUTION

DEAN
SCHOOL OF BIOSECURITY, BIOTECHNICAL & LABORATORY SCIENCES (SBLS)
14 DEC 2015
MAKERERE UNIVERSITY
P.O.BOX 7062, KAMPALA, UGANDA
