# Supplementary material for: The sero-prevalence of brucellosis in cattle and their herders in Bahr el Ghazal region, South Sudan
Source: PLoS Negl Trop Dis. 2018 Jun 20;12(6):e0006456. doi: 10.1371/journal.pntd.0006456 (PMC6010255; doi:10.1371/journal.pntd.0006456)
Supplement: S2 Ethical Approval — (PDF) [file pntd.0006456.s002.pdf]

The Republic of South Sudan

Ministry of Health

Promotes Health & Provides Care

Ministry of Health

21/04/2016

To: Nuol Aywel Madut Yaji
Makerere University
Collage of Veterinary Medicine, Animal Resources & Biosecurity

RESEARCH APPROVAL LETTER

Dear Madut,

SUBJECT: Brucellosis at Human- Domestic Animal Interface in Greater Bahr El Ghazal States, South Sudan

I am writing in response to the request for authorization for the study on "**Brucellosis at Human- Domestic Animal Interface in Greater Bahr El Ghazal States"** As part of your thesis.

After close review on the proposal, I am glad to inform you that the ethical committee at the Ministry of Health for the Republic of South Sudan has approved the study. The ministry acknowledges the importance of the study to provide useful data for intervention police on disease control.

Please, keep the Ministry of Health, Republic of South Sudan and States Ministry of Health of Lakes, Wau, Warrap and Aweil informed on the implementation progress. I look forward to the report and recommendations that will be generated from the study. Note that the study should not be published without the consent of the MOH-RSS.
Best regards.

for [signature]

MINISTRY OF HEALTH
21 APR 2016
REPUBLIC OF SOUTH SUDAN

Dr. Richard Lino Loro Lako
Director of Research, Monitoring and Evaluation
Ministry of Health, Republic of South Sudan, Juba

CC: Under Secretary, MOH-RSS
CC: Director General, preventive Health
CC: Director Generals, Greater Bahr El Ghazal States

Headquarters, Ministerial Complex. Juba, South Sudan - P.O.Box 88, Juba.
Tel: +211 (0) 177 800 281 / +211 (0) 177 800 278
