# Supplementary material for: The sero-prevalence of brucellosis in cattle and their herders in Bahr el Ghazal region, South Sudan
Source: PLoS Negl Trop Dis. 2018 Jun 20;12(6):e0006456. doi: 10.1371/journal.pntd.0006456 (PMC6010255; doi:10.1371/journal.pntd.0006456)
Supplement: S3 Ethical Approval — (PDF) [file pntd.0006456.s003.pdf]

The Republic of South Sudan

Healthy Livestock - Healthy People - Sustainable Development

Ministry of Livestock and Fisheries Industry
Directorate of Veterinary Services
Office of Director General

Ref: RSS/MLFI/DVS/J/15/7
Date: 6th December, 2016

To: Director Generals
State Ministry of Animal Resources and Fisheries
Directorate of Veterinary Services

Re: **Authorization for Dr. Noul Aywel Madut Yajj (PhD student) to collect samples (Animal and Human) in your States**

Dear Sir,

Reference to letter numbered SBLS.NA.2015 dated 14/12/2015 for the ethics committee form Makerere University. Collage of Veterinary Medicine, Animal Resources and Biosecurity. We would like to certify that **Dr. Noul Aywel Madut Yajj (*PhD Student*)** is doing a research in South Sudan for Brucellosis both in Animal and Human.

The PhD student has been working in the University of Bahr el Ghazal, Collage of Veterinary Science as lecture

We kindly request, therefore, you're esteemed Office to endorse this letter to facilitate the above-cited request to enable the PhD student to collect the following samples

Material

Bovine serum (1364)
Human serum (200)
Lymph node (330)
Swabs (330)
Milk (250)

These samples for testing in Kampala – Uganda to help activities for livestock disease control in the Country these samples are required for testing of Brucellosis.

Please accept the assurance of our highest regards.

For **Dr. Jacob M. Korok**

Acting Director General of Veterinary Services
Ministry of Livestock and Fisheries Industries
The Republic of South Sudan – Juba

MINISTRY OF LIVESTOCK AND FISHERIES INDUSTRIES
06 JAN 2016
OFFICE OF THE ADMINISTRATION

Phone. No. + 211 (0) 956 479 074 / 914 845 596 **Email:** jacobkorok@yahoo.co.uk
