# Supplementary material for: The sero-prevalence of brucellosis in cattle and their herders in Bahr el Ghazal region, South Sudan
Source: PLoS Negl Trop Dis. 2018 Jun 20;12(6):e0006456. doi: 10.1371/journal.pntd.0006456 (PMC6010255; doi:10.1371/journal.pntd.0006456)
Supplement: S4 Ethical Approval — (PDF) [file pntd.0006456.s004.pdf]

MINISTRY OF AGRICULTURE,
ANIMAL INDUSTRY AND FISHERIES
WEBSITE: www.agriculture.go.ug
DEPARTMENT OF LIVESTOCK HEALTH
AND ENTOMOLOGY
P. O. Box 513, ENTEBBE, UGANDA
E-MAIL: dlhe.maaif@imul.com
TELEPHONE: 256 041 320 627, 320166
FAX: 256-041-321047, 256-041-321010,
256-041-321255,320428

THE REPUBLIC OF UGANDA

In any correspondence on
this subject please quote **LHE 46/172/406**

30th October 2015

Noul Aywel Madut Yajj (PhD - STUDENT)
C/O
College of Veterinary Medicine
Animal Resources and Biosecurity
Makerere University
P.O. Box 7062,
KAMPALA - UGANDA

RE: IMPORT PERMIT FOR IMPORTATION OF SERUM WHOLE BLOOD HUMANS AND CATTLE FROM SOUTH SUDAN INTO UGANDA

Your request of 14th October 2015 refers; Permission is granted to you to import Serum whole blood humans and cattle for bovine Tuberculosis Research and Training purposes from South Sudan to Makerere University Kampala, Uganda.

The following conditions must be met by the importer;

1. The samples must be handled in a laboratory (or laboratory suite) that operates at containment level 2 as defined by the OIE (OIE Terrestrial Manual 2008, Chapter 1.1.2 Biosafety and Biosecurity in the veterinary Microbiology Laboratory and Animal facilities.

2. The importer must destroy all received biological samples at the end of the study under supervision of the Commissioner Animal Health accordance to requirements for dangerous goods transportation.

3. The importer must confirm that the samples above will neither be used in any form or way for purposes associated with chemical, biological weapon nor will they be re- exported, resold or otherwise transmitted to another institute.

30/10/15

COMMISSIONER ANIMAL HEALTH
MINISTRY OF AGRICULTURE,
ANIMAL INDUSTRY AND FISHERIES
P. O. Box 513, Entebbe - Uganda

4. The sample should be packaged in such away that there will be no leakage during transportation.

5. This import permission is valid for a period of one month from date of issue and is subject to cancellation should conditions necessitating doing so arise.

COMMISSIONER ANIMAL HEALTH
MINISTRY OF AGRICULTURE,
ANIMAL INDUSTRY AND FISHERIES
P. O. Box 513, Entebbe - Uganda

Kasirye Martin (Dr)
For: COMMISSIONER ANIMAL HEALTH

c.c.: Asst. Commissioner Veterinary Inspections and Regulations
" Customs officer (EIA)
" Senior Veterinary Inspector – EIA
" PVO Diagnostic and Epidemiology

Tel: +256- 778-893284, 782-334637, 772-440994
Email: dvdnsuga@yahoo.co.uk, nanozibeatrice@yahoo.com
