# Supplementary material for: The sero-prevalence of brucellosis in cattle and their herders in Bahr el Ghazal region, South Sudan
Source: PLoS Negl Trop Dis. 2018 Jun 20;12(6):e0006456. doi: 10.1371/journal.pntd.0006456 (PMC6010255; doi:10.1371/journal.pntd.0006456)
Supplement: S3 Dataset — (HTML) [file pntd.0006456.s008.html]

Model summaries


# Model summaries

#### *AM*

#### *12 January 2017*

## ••••••••••••••••••••••••••••••••••••••••••••••••••••••••••••••••••••••••••••••••••••••••••••••••••••••••••••••••••••••••••

### DATA ANALYSIS: Bovine and Human Brucellosis Sero-prevalence and Associated Factors in Bahr el Ghazal region, South Sudan

# ••••••••••••••••••••••••••••••••••••••••••••••••••••••••••••••••••••••••••••••••••••••••••••••••••••••••••••••••••••••••••••

```
library(Amelia)
library(lmtest)
library(ResourceSelection)
library(MKmisc)
library("AUC") 
library(LogisticDx)
library(vcd)
library(pROC)
library("elrm")
library(dplyr)
library(foreign)
library(data.table)
library(dplyr)


# Loading the Cattle dataset
DataDB_BRUCE<-read.spss("/Users/amuwonge/Dropbox/capazomaniteco/Archive/SUPERVISION/Southern Sudan/Noul_Brucella/data base/BRUCELLA_ROSS.sav",to.data.frame = T)
DataDB_HUM<- read.spss("/Users/amuwonge/Dropbox/capazomaniteco/Archive/SUPERVISION/Southern Sudan/Noul_Brucella/data base/TonjHumanField-2.sav",to.data.frame = T)
missmap(DataDB_HUM, main = "Missing values vs observed")
```

```
#•••••••••••••••••••••••••••••••••
##Diagnostic test agreement
#•••••••••••••••••••••••••••••••••

table(DataDB_BRUCE$rbpt_rec,DataDB_BRUCE$cElisa_rec)
```

```
##           
##            Negative Positive
##   Negative      611        1
##   Positive        5      276
```

```
# Univariate data exploration
table(DataDB_BRUCE$cElisa_rec, DataDB_BRUCE$state)
```

```
##           
##            WAU GOGRIAL TONJ AWIEL
##   Negative  51      63  133   369
##   Positive  87       7   65   118
```

```
table(DataDB_BRUCE$rbpt_rec, DataDB_BRUCE$state)
```

```
##           
##            WAU GOGRIAL TONJ AWIEL
##   Negative  47      63  133   369
##   Positive  91       7   65   118
```

```
table(DataDB_BRUCE$cElisa_rec, DataDB_BRUCE$locality)
```

```
##           
##            Rojroj Dong Guarjina Kuajok Hi Mango Auction Abada West Nyamae
##   Negative          47        4     63       78     187         30    103
##   Positive          60       27      7       41      46         10     24
##           
##            Awada Kwangi Waremeal
##   Negative    49     40       15
##   Positive    38     12       12
```

```
table(DataDB_BRUCE$rbpt_rec, DataDB_BRUCE$locality)
```

```
##           
##            Rojroj Dong Guarjina Kuajok Hi Mango Auction Abada West Nyamae
##   Negative          43        4     63       78     187         30    103
##   Positive          64       27      7       41      46         10     24
##           
##            Awada Kwangi Waremeal
##   Negative    49     40       15
##   Positive    38     12       12
```

```
table(DataDB_BRUCE$cElisa_rec, DataDB_BRUCE$locality)
```

```
##           
##            Rojroj Dong Guarjina Kuajok Hi Mango Auction Abada West Nyamae
##   Negative          47        4     63       78     187         30    103
##   Positive          60       27      7       41      46         10     24
##           
##            Awada Kwangi Waremeal
##   Negative    49     40       15
##   Positive    38     12       12
```

```
table(DataDB_BRUCE$rbpt_rec, DataDB_BRUCE$locality)
```

```
##           
##            Rojroj Dong Guarjina Kuajok Hi Mango Auction Abada West Nyamae
##   Negative          43        4     63       78     187         30    103
##   Positive          64       27      7       41      46         10     24
##           
##            Awada Kwangi Waremeal
##   Negative    49     40       15
##   Positive    38     12       12
```

```
table(DataDB_BRUCE$cElisa_rec, DataDB_BRUCE$age_group)
```

```
##           
##            0 - 5 6 - 10 11 - 20
##   Negative   207    300     109
##   Positive    76    113      88
```

```
table(DataDB_BRUCE$rbpt_rec, DataDB_BRUCE$age_group)
```

```
##           
##            0 - 5 6 - 10 11 - 20
##   Negative   207    299     106
##   Positive    76    114      91
```

```
table(DataDB_BRUCE$cElisa_rec, DataDB_BRUCE$sex)
```

```
##           
##             F   M 
##   Negative 450 166
##   Positive 196  81
```

```
table(DataDB_BRUCE$rbpt_rec, DataDB_BRUCE$sex)
```

```
##           
##             F   M 
##   Negative 448 164
##   Positive 198  83
```

```
table(DataDB_BRUCE$cElisa_rec, DataDB_BRUCE$herd_size_rec)
```

```
##           
##            < 30 > 100 30-49 50-100
##   Negative    4   545    47     20
##   Positive    6   165    81     25
```

```
table(DataDB_BRUCE$rbpt_rec, DataDB_BRUCE$herd_size_rec)
```

```
##           
##            < 30 > 100 30-49 50-100
##   Negative    3   545    44     20
##   Positive    7   165    84     25
```

```
table(DataDB_BRUCE$cElisa_rec, DataDB_BRUCE$lactating_stage_rec)
```

```
##           
##            DRY  L1  L2  L3
##   Negative 263  32 109  46
##   Positive  90  10  56  40
```

```
table(DataDB_BRUCE$rbpt_rec, DataDB_BRUCE$lactating_stage_rec)
```

```
##           
##            DRY  L1  L2  L3
##   Negative 262  32 109  45
##   Positive  91  10  56  41
```

```
table(DataDB_BRUCE$cElisa_rec, DataDB_BRUCE$health_status_rec) #This represents body condition rather than health status
```

```
##           
##            GOOD POOR WEAK
##   Negative  555   52    9
##   Positive  261    5   11
```

```
table(DataDB_BRUCE$rbpt_rec, DataDB_BRUCE$health_status_rec) #This represents body condition rather than health status
```

```
##           
##            GOOD POOR WEAK
##   Negative  552   52    8
##   Positive  264    5   12
```

```
table(DataDB_BRUCE$cElisa_rec, DataDB_BRUCE$hygroma_sample_rec)
```

```
##           
##             No Yes
##   Negative 613   2
##   Positive 269   7
```

```
table(DataDB_BRUCE$rbpt_rec, DataDB_BRUCE$hygroma_sample_rec)
```

```
##           
##             No Yes
##   Negative 609   2
##   Positive 273   7
```

```
table(DataDB_BRUCE$cElisa_rec, DataDB_BRUCE$history_of_abortion_rec)
```

```
##           
##             No Yes
##   Negative 553  14
##   Positive 192  15
```

```
table(DataDB_BRUCE$rbpt_rec, DataDB_BRUCE$history_of_abortion_rec)
```

```
##           
##             No Yes
##   Negative 553  14
##   Positive 192  15
```

```
table(DataDB_BRUCE$health_status_rec, DataDB_BRUCE$state)
```

```
##       
##        WAU GOGRIAL TONJ AWIEL
##   GOOD 118      19  192   487
##   POOR   0      51    6     0
##   WEAK  20       0    0     0
```

```
table(DataDB_BRUCE$history_of_abortion_rec, DataDB_BRUCE$state)
```

```
##      
##       WAU GOGRIAL TONJ AWIEL
##   No   10      60  188   487
##   Yes  20       2    7     0
```

```
table(DataDB_BRUCE$hygroma_sample_rec, DataDB_BRUCE$state)
```

```
##      
##       WAU GOGRIAL TONJ AWIEL
##   No  133      68  194   487
##   Yes   5       0    4     0
```

```
# Chisq test for potential model variable
chisq.test(DataDB_BRUCE$cElisa_rec,DataDB_BRUCE$state)
```

```
## 
##  Pearson's Chi-squared test
## 
## data:  DataDB_BRUCE$cElisa_rec and DataDB_BRUCE$state
## X-squared = 91.39, df = 3, p-value < 2.2e-16
```

```
chisq.test(DataDB_BRUCE$cElisa_rec,DataDB_BRUCE$milk_sample_rec)
```

```
## 
##  Pearson's Chi-squared test with Yates' continuity correction
## 
## data:  DataDB_BRUCE$cElisa_rec and DataDB_BRUCE$milk_sample_rec
## X-squared = 3.0295, df = 1, p-value = 0.08176
```

```
chisq.test(DataDB_BRUCE$cElisa_rec,DataDB_BRUCE$swab_sample_rec)
```

```
## 
##  Pearson's Chi-squared test with Yates' continuity correction
## 
## data:  DataDB_BRUCE$cElisa_rec and DataDB_BRUCE$swab_sample_rec
## X-squared = 0.68752, df = 1, p-value = 0.407
```

```
chisq.test(DataDB_BRUCE$cElisa_rec,DataDB_BRUCE$hygroma_sample_rec)# two rows without hygroma info hense the error in chisq test
```

```
## 
##  Pearson's Chi-squared test with Yates' continuity correction
## 
## data:  DataDB_BRUCE$cElisa_rec and DataDB_BRUCE$hygroma_sample_rec
## X-squared = 7.2341, df = 1, p-value = 0.007153
```

```
chisq.test(DataDB_BRUCE$cElisa_rec,DataDB_BRUCE$health_status_rec)# This represents body condition rather than health status
```

```
## 
##  Pearson's Chi-squared test
## 
## data:  DataDB_BRUCE$cElisa_rec and DataDB_BRUCE$health_status_rec
## X-squared = 18.916, df = 2, p-value = 7.807e-05
```

```
chisq.test(DataDB_BRUCE$cElisa_rec,DataDB_BRUCE$history_of_abortion_rec)
```

```
## 
##  Pearson's Chi-squared test with Yates' continuity correction
## 
## data:  DataDB_BRUCE$cElisa_rec and DataDB_BRUCE$history_of_abortion_rec
## X-squared = 8.3171, df = 1, p-value = 0.003927
```

```
chisq.test(DataDB_BRUCE$cElisa_rec,DataDB_BRUCE$lactating_stage_rec)
```

```
## 
##  Pearson's Chi-squared test
## 
## data:  DataDB_BRUCE$cElisa_rec and DataDB_BRUCE$lactating_stage_rec
## X-squared = 16.42, df = 3, p-value = 0.00093
```

```
chisq.test(DataDB_BRUCE$cElisa_rec,DataDB_BRUCE$sex_rec)
```

```
## 
##  Pearson's Chi-squared test with Yates' continuity correction
## 
## data:  DataDB_BRUCE$cElisa_rec and DataDB_BRUCE$sex_rec
## X-squared = 0.39436, df = 1, p-value = 0.53
```

```
chisq.test(DataDB_BRUCE$cElisa_rec,DataDB_BRUCE$age_group)
```

```
## 
##  Pearson's Chi-squared test
## 
## data:  DataDB_BRUCE$cElisa_rec and DataDB_BRUCE$age_group
## X-squared = 22.033, df = 2, p-value = 1.643e-05
```

```
chisq.test(DataDB_BRUCE$cElisa_rec,DataDB_BRUCE$herd_size_rec)## number of categories do not allow for chisq approximation
```

```
## 
##  Pearson's Chi-squared test
## 
## data:  DataDB_BRUCE$cElisa_rec and DataDB_BRUCE$herd_size_rec
## X-squared = 98.934, df = 3, p-value < 2.2e-16
```

```
table(DataDB_BRUCE$state,DataDB_BRUCE$herd_size_rec) ### Can only use one these in the model
```

```
##          
##           < 30 > 100 30-49 50-100
##   WAU       10     0   128      0
##   GOGRIAL    0    70     0      0
##   TONJ       0   153     0     45
##   AWIEL      0   487     0      0
```

```
### check for db completeness
missmap(DataDB_BRUCE, main = "Missing values vs observed")
```

```
## For ther regression model we shall only keep a complete data abase
xx<-DataDB_BRUCE[complete.cases(DataDB_BRUCE$lactating_stage_rec),]
xxx<-xx[complete.cases(xx$herd_size_rec),]
xxxx<-xxx[complete.cases(xxx$history_of_abortion_rec),]
xxxxx<-xxxx[complete.cases(xxxx$cElisa_rec),]
DataDB_BRUCE.complete<-xxxxx

missmap(DataDB_BRUCE.complete, main = "Missing values vs observed")
```

```
DataDB_BRUCE_MOD<- DataDB_BRUCE.complete[,c(10,14:17,20,24)] ### Select only variable that are in the Model
missmap(DataDB_BRUCE_MOD, main = "Missing values vs observed")
```

```
#Logistic regression model selection
DataDB_BRUCE_MOD$herd_size_rec<- factor(DataDB_BRUCE_MOD$herd_size_rec, level=c(" < 30 ", "30-49", "50-100","> 100"))
model<-glm(cElisa_rec ~ as.factor(lactating_stage_rec)+ as.factor(herd_size_rec) + as.factor(history_of_abortion_rec) + as.factor(hygroma_sample_rec) ,family=binomial(link='logit'),data=DataDB_BRUCE_MOD)
summary(model)
```

```
## 
## Call:
## glm(formula = cElisa_rec ~ as.factor(lactating_stage_rec) + as.factor(herd_size_rec) + 
##     as.factor(history_of_abortion_rec) + as.factor(hygroma_sample_rec), 
##     family = binomial(link = "logit"), data = DataDB_BRUCE_MOD)
## 
## Deviance Residuals: 
##     Min       1Q   Median       3Q      Max  
## -1.6741  -0.7893  -0.6763   0.9619   2.2088  
## 
## Coefficients:
##                                       Estimate Std. Error z value Pr(>|z|)
## (Intercept)                            1.22083    0.61762   1.977  0.04808
## as.factor(lactating_stage_rec)L1      -0.07743    0.41687  -0.186  0.85265
## as.factor(lactating_stage_rec)L2       0.35225    0.22536   1.563  0.11804
## as.factor(lactating_stage_rec)L3       0.88670    0.27666   3.205  0.00135
## as.factor(herd_size_rec)50-100        -1.06000    0.66833  -1.586  0.11273
## as.factor(herd_size_rec)> 100         -2.57981    0.62329  -4.139 3.49e-05
## as.factor(history_of_abortion_rec)Yes -0.98903    0.65222  -1.516  0.12942
## as.factor(hygroma_sample_rec)Yes       0.94463    1.00682   0.938  0.34812
##                                          
## (Intercept)                           *  
## as.factor(lactating_stage_rec)L1         
## as.factor(lactating_stage_rec)L2         
## as.factor(lactating_stage_rec)L3      ** 
## as.factor(herd_size_rec)50-100           
## as.factor(herd_size_rec)> 100         ***
## as.factor(history_of_abortion_rec)Yes    
## as.factor(hygroma_sample_rec)Yes         
## ---
## Signif. codes:  0 '***' 0.001 '**' 0.01 '*' 0.05 '.' 0.1 ' ' 1
## 
## (Dispersion parameter for binomial family taken to be 1)
## 
##     Null deviance: 724.84  on 605  degrees of freedom
## Residual deviance: 669.11  on 598  degrees of freedom
## AIC: 685.11
## 
## Number of Fisher Scoring iterations: 4
```

```
exp(cbind(Odds_and_OR=coef(model), confint(model)))
```

```
##                                       Odds_and_OR      2.5 %     97.5 %
## (Intercept)                            3.38998456 1.09975654 13.1173783
## as.factor(lactating_stage_rec)L1       0.92549145 0.38759256  2.0189606
## as.factor(lactating_stage_rec)L2       1.42226570 0.91081564  2.2068264
## as.factor(lactating_stage_rec)L3       2.42711060 1.40411703  4.1660304
## as.factor(herd_size_rec)50-100         0.34645544 0.08360947  1.2100812
## as.factor(herd_size_rec)> 100          0.07578815 0.01941873  0.2363958
## as.factor(history_of_abortion_rec)Yes  0.37193732 0.09181402  1.2414730
## as.factor(hygroma_sample_rec)Yes       2.57185961 0.34426162 22.1352406
```

```
## model fit validation 

hoslem.test(DataDB_BRUCE_MOD$cElisa_rec, fitted(model), g=10) ## Although the fit analysis does not converge, we still believe the model explains the dynamics in the field. This is likely because our sampling was done during a period of instability in Southern Sudan
```

```
## 
##  Hosmer and Lemeshow goodness of fit (GOF) test
## 
## data:  DataDB_BRUCE_MOD$cElisa_rec, fitted(model)
## X-squared = 606, df = 8, p-value < 2.2e-16
```

```
prob<-predict(model,type=c("response"),newdata = DataDB_BRUCE_MOD)
DataDB_BRUCE_MOD$prob<-prob
p<- roc(cElisa_rec~prob,data=DataDB_BRUCE_MOD)
plot(p)#### The model explains ~ 65%
```

```
## 
## Call:
## roc.formula(formula = cElisa_rec ~ prob, data = DataDB_BRUCE_MOD)
## 
## Data: prob in 433 controls (cElisa_rec Negative) < 173 cases (cElisa_rec Positive).
## Area under the curve: 0.6505
```

```
plot(ci.thresholds(p))
```

``` ################################################################################################## # DATA ANALYSIS OF HUMAN BRUCELLOSIS DATA SOUTHERN SUDAN ##################################################################################################

```
table(DataDB_HUM$sex)
```

```
## 
##   Male Female 
##     25     62
```

```
table(DataDB_HUM$CELISA_rec)
```

```
## 
## NEGATIVE POSITIVE 
##       58       29
```

```
table(DataDB_HUM$age_group)
```

```
## 
##   0-5  6-15 16-35 36-60   >61 
##     2    13    49    21     2
```

```
table(DataDB_HUM$location_rec)
```

```
## 
##  AUCTION Hi mango   KUANGI    NYMAE WAREMAEL 
##       29       15       20       11       12
```

```
DataDB_HUM$age_groupI<-NA

DataDB_HUM$age_groupI[DataDB_HUM$age_group=="6-15"]<-"6-15"
DataDB_HUM$age_groupI[DataDB_HUM$age_group=="0-5"]<-"6-15"

DataDB_HUM$age_groupI[DataDB_HUM$age_group=="36-60"]<-"36-60"
DataDB_HUM$age_groupI[DataDB_HUM$age_group==">61"]<-"36-60"

DataDB_HUM$age_groupI[DataDB_HUM$age_group=="16-35"]<-"16-35"

table(DataDB_HUM$SAT_rec)
```

```
## 
## NEGATIVE POSITIVE 
##       48       39
```

```
chisq.test(DataDB_HUM$CELISA_rec, DataDB_HUM$sex)
```

```
## 
##  Pearson's Chi-squared test with Yates' continuity correction
## 
## data:  DataDB_HUM$CELISA_rec and DataDB_HUM$sex
## X-squared = 1.1857, df = 1, p-value = 0.2762
```

```
chisq.test(DataDB_HUM$CELISA_rec, DataDB_HUM$age_groupI)
```

```
## 
##  Pearson's Chi-squared test
## 
## data:  DataDB_HUM$CELISA_rec and DataDB_HUM$age_groupI
## X-squared = 2.2754, df = 2, p-value = 0.3206
```

```
chisq.test(DataDB_HUM$CELISA_rec, DataDB_HUM$location_rec)
```

```
## 
##  Pearson's Chi-squared test
## 
## data:  DataDB_HUM$CELISA_rec and DataDB_HUM$location_rec
## X-squared = 6.3877, df = 4, p-value = 0.172
```

```
chisq.test(DataDB_HUM$CELISA_rec, DataDB_HUM$occupation_rec)
```

```
## 
##  Pearson's Chi-squared test with Yates' continuity correction
## 
## data:  DataDB_HUM$CELISA_rec and DataDB_HUM$occupation_rec
## X-squared = 0.011352, df = 1, p-value = 0.9152
```

```
HUMAN_sum<-summarise(group_by(DataDB_HUM,occupation_rec,age_groupI,location_rec,sex,occupation_rec),success=sum(CELISA_rec=="POSITIVE"),trials=length(CELISA_rec))
HUMAN_sum$prob_bruce<- (HUMAN_sum$success/HUMAN_sum$trials)*100
HUMAN_sum<-HUMAN_sum[!HUMAN_sum$prob_bruce<=0,]
```

Fitting generalized (binomial/logit) linear model: cElisa\_rec ~ as.factor(lactating\_stage\_rec) + as.factor(herd\_size\_rec) + as.factor(history\_of\_abortion\_rec) + as.factor(hygroma\_sample\_rec) (continued below)


|  | Estimate | Std. Error | z value |
| --- | --- | --- | --- |
| **as.factor(lactating\_stage\_rec)L1** | -0.07743 | 0.4169 | -0.1857 |
| **as.factor(lactating\_stage\_rec)L2** | 0.3523 | 0.2254 | 1.563 |
| **as.factor(lactating\_stage\_rec)L3** | 0.8867 | 0.2767 | 3.205 |
| **as.factor(herd\_size\_rec)50-100** | -1.06 | 0.6683 | -1.586 |
| **as.factor(herd\_size\_rec)> 100** | -2.58 | 0.6233 | -4.139 |
| **as.factor(history\_of\_abortion\_rec)Yes** | -0.989 | 0.6522 | -1.516 |
| **as.factor(hygroma\_sample\_rec)Yes** | 0.9446 | 1.007 | 0.9382 |
| **(Intercept)** | 1.221 | 0.6176 | 1.977 |

|  | Pr(>|z|) |
| --- | --- |
| **as.factor(lactating\_stage\_rec)L1** | 0.8526 |
| **as.factor(lactating\_stage\_rec)L2** | 0.118 |
| **as.factor(lactating\_stage\_rec)L3** | 0.00135 |
| **as.factor(herd\_size\_rec)50-100** | 0.1127 |
| **as.factor(herd\_size\_rec)> 100** | 3.487e-05 |
| **as.factor(history\_of\_abortion\_rec)Yes** | 0.1294 |
| **as.factor(hygroma\_sample\_rec)Yes** | 0.3481 |
| **(Intercept)** | 0.04808 |

| occupation\_rec | age\_groupI | location\_rec | sex | success | trials | prob\_bruce |
| --- | --- | --- | --- | --- | --- | --- |
| Farmer | 16-35 | AUCTION | Male | 1 | 3 | 33.33333 |
| Farmer | 16-35 | AUCTION | Female | 2 | 5 | 40.00000 |
| Farmer | 16-35 | Hi mango | Male | 1 | 3 | 33.33333 |
| Farmer | 16-35 | Hi mango | Female | 2 | 5 | 40.00000 |
| Farmer | 16-35 | NYMAE | Male | 1 | 2 | 50.00000 |
| Farmer | 36-60 | NYMAE | Male | 5 | 5 | 100.00000 |
| Milking | 16-35 | AUCTION | Female | 4 | 8 | 50.00000 |
| Milking | 16-35 | KUANGI | Female | 4 | 12 | 33.33333 |
| Milking | 16-35 | WAREMAEL | Female | 1 | 7 | 14.28571 |
| Milking | 36-60 | AUCTION | Female | 1 | 1 | 100.00000 |
| Milking | 36-60 | KUANGI | Male | 1 | 1 | 100.00000 |
| Milking | 36-60 | NYMAE | Female | 1 | 1 | 100.00000 |
| Milking | 36-60 | WAREMAEL | Female | 1 | 1 | 100.00000 |
| Milking | 6-15 | AUCTION | Female | 1 | 4 | 25.00000 |
| Milking | 6-15 | WAREMAEL | Male | 1 | 1 | 100.00000 |
| NA | 36-60 | WAREMAEL | Male | 1 | 1 | 100.00000 |
| NA | 6-15 | AUCTION | Female | 1 | 2 | 50.00000 |
